# Serum Dynamin-Related Protein 1 Concentrations Discriminate Phenotypes and Predict Prognosis of Heart Failure

**DOI:** 10.31083/j.rcm2404123

**Published:** 2023-04-23

**Authors:** Zhong-guo Fan, Ming-yue Ji, Yang Xu, Wan-xin Wang, Jing Lu, Gen-Shan Ma

**Affiliations:** ^1^Department of Cardiology, Zhongda Hospital, School of Medicine, Southeast University, 210009 Nanjing, Jiangsu, China; ^2^Department of Cardiology, Lianshui People’s Hospital, 223400 Huaian, Jiangsu, China

**Keywords:** dynamin-related protein 1, heart failure, mitochondria, diagnosis, prognosis, mitophagy

## Abstract

**Background:**

Dynamin-related protein 1 (Drp1) has been demonstrated as a crucial role in mediating the programed cell death and cardiac metabolism through its regulatory of mitophagy in animal studies. However, the clinical values of Drp1 for human cardiac disease remain unknown. This study is aimed to evaluate the diagnostic and prognostic values of serum Drp1 in these patients with heart failure (HF).

**Methods:**

The enzyme linked immunosorbent assay (ELISA) was used for measuring serum Drp1 concentrations in 85 cases of HF with preserved ejection fraction (HFpEF) and 86 cases of HF with reduced ejection fraction (HFrEF). The diagnostic value of Drp1 was evaluated using the receiver operating characteristic (ROC) analysis. The composite endpoint was consisted of cardiac death and rehospitalization for HF, and the association between Drp1 and clinical outcomes were further determined.

**Results:**

Serum Drp1 concentrations were much higher in HFpEF than that in HFrEF (4.2 ± 3.7 ng/mL vs. 2.6 ± 2.2 ng/mL, *p* = 0.001) and the ROC analysis demonstrated it as a potential diagnostic biomarker for distinction of the HF phenotypes, with an optimal cutoff point of 3.5 ng/mL (area under the curve (AUC) = 0.659, sensitivity: 45.9%, specificity: 83.7%). Kaplan-Meier survival analysis indicated that a low serum concentration of Drp1 (cut-off value = 2.5 ng/mL, AUC = 0.738) was in relation to poor prognosis of HF. Moreover, binary logistic regression analysis identified the low serum concentration of Drp1 as an independent risk predictor for rehospitalization (odds ratio (OR) = 6.574, *p* = 0.001) and a composite endpoint (OR = 5.927, *p* = 0.001).

**Conclusions:**

Our findings suggested that low serum concentrations of Drp1 might serve as a predicting biomarker for distinction of HF phenotypes and overall prognosis of HF.

## 1. Introduction

Heart failure (HF) is a manifestation of cardiac dysfunction secondary to abnormalities in cardiac structure, which progress to a state of decompensation and then fail to keep up with the metabolic needs of the body [1]. With the growing numbers of elderly populations and increased incidence of risk factors [e.g., coronary artery disease (CAD), hypertension, diabetes mellitus (DM), obesity, and smoking], the prevalence of HF is rapidly rising, leading to increasing medical and socioeconomic burdens world-wide [2, 3]. A prior report showed a 12-month mortality rate of 16.5% for HF and the absolute mortality rate within 5 years after a diagnosis of HF may reach approximately 50% [4]. HF has been classified into different phenotypes to help guide the clinical management for this disease. The survival and hospitalization rate of HF with reduced ejection fraction (HFrEF) has benefited from the development of medical therapies and cardiac assist equipment [5]. Once HF with preserved ejection fraction (HFpEF) occurs, the typical dyspnea symptoms manifested in HFrEF will not appear because this phenotype of HF is characterized by restricted filling and disturbed relaxation of the myocardium whereas the systolic function is close to normal. The risk of this specific subset of HF is not fully understood [6]. Hence, the overall prognosis for HF still remains unsatisfactory. There have been numerous biomarkers for the diagnosis of HF [7], but the pathophysiology regarding the progression and evolution of this disease still needs to be further elucidated. Therefore, it is important to investigate new potential diagnostic and therapeutic biomarkers for these patients, especially for those with HFpEF.

The heart is the most metabolically active organ in the human body, and it accounts for approximately 8% of daily ATP consumption [8]. Mitochondria act as the powerhouse of the cells and are responsible for normal cell metabolism, protecting cells against damage from reactive oxygen species (ROS) [9]. Dynamin-related protein 1 (Drp1) belongs to the dynamin family of GTP-binding proteins. They often translocate from the cytoplasm to the mitochondria and then bind to their targets located in the outer mitochondrial membrane (OMM) to induce mitochondrial fission, thereby mediating mitophagy to affect programmed cell death and cell metabolism [10, 11, 12]. Drp1 can be expressed as multiple splice variants, which are highly expressed in the human heart, skeletal muscle, brain, and kidney [13, 14]. Several studies have demonstrated the association between mitochondrial bioenergetic capacity and progression of HF, in which impaired mitochondrial energetics greatly contributed to the onset and progression of maladaptive cardiac hypertrophy [15, 16]. Therefore, there may be a potential association between Drp1 and HF. This study was undertaken to explore the role of serum Drp1 in HF patients, especially in those with HFpEF.

## 2. Methods

### 2.1 Study Population

From September 2021 to April 2022, patients hospitalized at the Zhongda Hospital (Nanjing, China) were consecutively enrolled in this prospective, single-center, observational study according to the following inclusion criteria: (1) adult patients (aged from 18 to 85 years) who were diagnosed with HF for at least 3 months, and (2) had good compliance with medical therapies. HF was diagnosed by at least two experienced cardiologists. The criteria for the diagnosis of HF were based on the presence of New York Heart Association (NYHA) classes II–IV symptoms, combined with abnormalities in cardiac structure on echocardiography and plasma levels of N-terminal pro–B-type natriuretic peptide (NT-proBNP) of at least 300 pg per milliliter. Echocardiography was performed the next day after admission. These patients were further divided based on echocardiography into the HFpEF (EF ≥50%) and HFrEF (EF <50%) subgroups. Exclusion criteria included: (1) a diagnosis of new onset HF; (2) a diagnosis of an acute myocardial infarction (AMI <24 h), macrovascular diseases (including acute aortic dissection, aortic stenosis or aorto-arteritis), congenital heart diseases, lung diseases, peripheral vascular diseases, pericardial diseases, myocarditis, heart valvular diseases, shock, thyroid diseases, or severe infection; (3) the presence of severe liver dysfunction (serum aspartate aminotransferase or alanine aminotransferase >140 U/L) or renal dysfunction (eGFR <30 mL/min/1.73 $m^{2}$); (4) refusal of enrollment or violation of the study protocol. The ethics committee of Zhongda Hospital approved the study protocol and informed consent (No. 2020ZDSYLL306-P01). All participants in the study provided written informed consent.

### 2.2 Plasma Collection and Serum Drp1 Measurements

Peripheral fasting blood (3–5 mL) was collected from all participants the next morning after admission. The blood samples were temporarily maintained at 4 °C and then centrifuged at 3000 r/min for 30 minutes. Next, the supernatant was collected into 1.5-mL EP tubes and stored at –80 °C until further measurements were made. Enzyme-linked immunosorbent assay (ELISA) kits (EH14381, FineTest, Wuhan, China) were used to detect the serum Drp1 concentrations in accordance with the manufacturer’s instructions, and all ELISA data were analyzed in relation to the standard curve.

### 2.3 Study Endpoint and Relevant Definitions

Clinical follow-up was conducted using telephone contact or clinical office visits at 1 month and 6 months after discharge. The composite endpoint of this study was cardiac death and rehospitalization for HF. An independent cardiologist assessed and recorded the relevant clinical events. Cardiac death refers to a death in the absence of non-cardiac causes confirmed by clinical or autopsy findings. To identify rehospitalization for HF, the electronic medical records of Zhongda Hospital were carefully screened, and patients or family members were interviewed if they were readmitted to other hospitals.

### 2.4 Statistical Analysis

All statistical analyses were performed using SPSS Statistics software, version 23.0 (SPSS Inc., Chicago, IL, USA). The Shapiro-Wilk test was first performed to determine the normality of continuous data. Normally distributed variables were recorded as mean ± standard deviation (SD), and Student’s *t*-tests were applied for comparisons between two groups. These non-normally distributed data were presented as the median with interquartile range (IQR) and compared using the Mann–Whitney U-test. Categorical variables were expressed as counts with percentages, and the chi-square test or Fisher exact test was used to compare differences between two groups. Comparisons between multiple groups were conducted via one-way analysis of variance with a post hoc Bonferroni correction in cases of equal variance, while the post hoc Tamhane test was used in cases of unequal variance. To explore the diagnostic ability of serum Drp1 and its relationship to the composite endpoint, receiver operating characteristic (ROC) curves were generated, and the optimal cut-off points were identified by the Youden index, respectively. Then, participants were classified into the low and high Drp1 groups based on the optimal cut-off point. Kaplan-Meier (K-M) analysis was utilized for generating the time-to-first event curves in the two groups, and the log-rank test was performed to compare their differences. Binary logistic regression was employed to examine whether serum Drp1 was independently associated with clinical endpoints after adjusting for potential confounding factors. Survival curves and the forest plot showing the results of the binary logistic regression were acquired using GraphPad Prism 8 (GraphPad Software, San Diego, CA, USA). A *p* value < 0.05 was considered as statistically significant, and all *p* values were two tailed.

## 3. Results

### 3.1 Baseline Characteristics of the Enrolled Populations

**Fig. 1.**
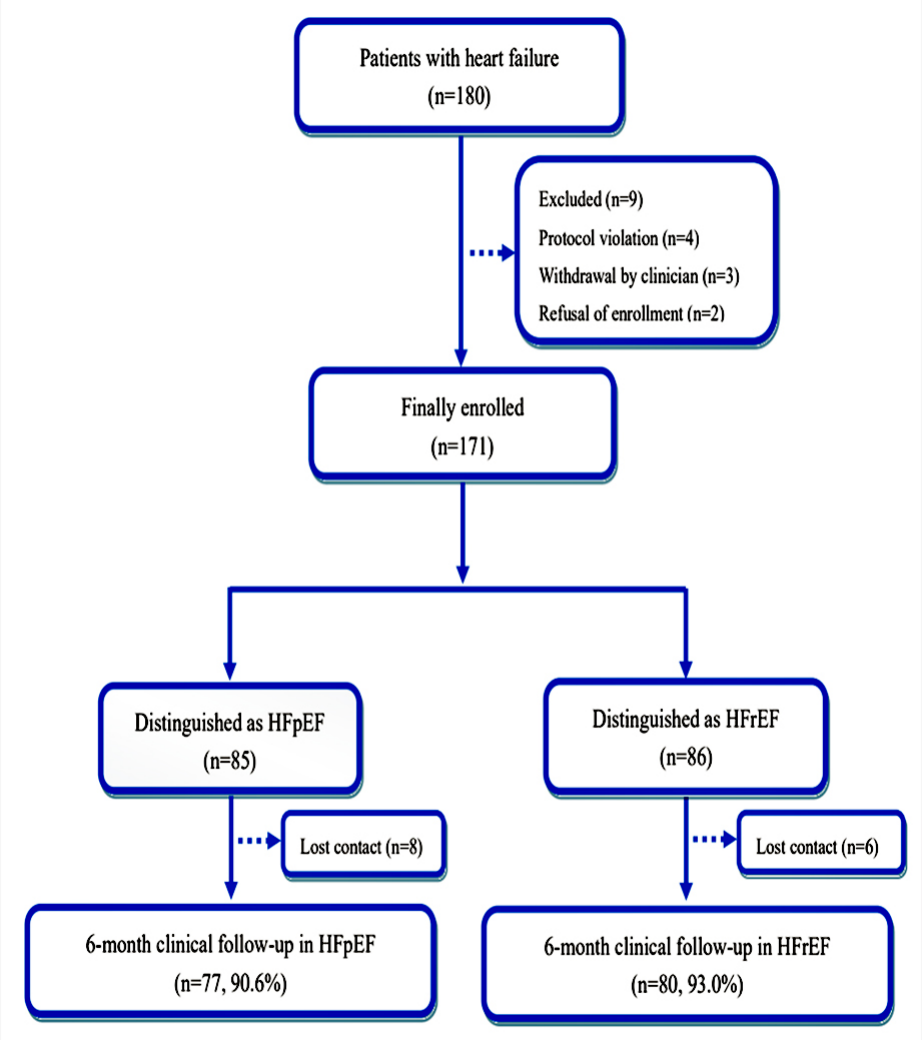
**A flow chart of the patients in this study**.

A total of 171 patients were enrolled from the Zhongda Hospital, including 85 patients with HFpEF and 86 patients with HFrEF. The majority of participants finished the 6-month follow-up and only 8.2% of patients were lost to follow up (Fig. 1). The baseline characteristics of these patients are summarized in Table 1. The etiology of HF was mainly from ischemic heart disease (IHD, 74.3%), especially secondary to a prior MI (53.2%), which was also the leading cause of HFrEF (61.6%). Compared to patients with HFrEF, patients with HFpEF were more likely to be females, older, and had an increased incidence of atrial fibrillation (AF) and hypertension. Plasma NT-proBNP levels were significantly higher in patients with HFrEF than in patients with HFpEF (3135.0 vs. 1290.0, *p *< 0.001). Echocardiography results differed significantly between the two groups. The remaining demographics and laboratory results were well matched between the two groups.

**Table 1.** **Baseline characteristics in HF patients**.

Variables		Total (n = 171)	HFpEF (n = 85)	HFrEF (n = 86)	*p*-value
Demographics					
	Male, n (%)	103 (60.2)	41 (48.2)	62 (72.1)	0.002
	Age, years	70.1 ± 11.4	72.1 ± 9.7	68.1 ± 12.6	0.021
	BMI, kg/$m^{2}$	25.8 ± 4.8	26.0 ± 4.3	25.7 ± 5.4	0.690
	Heart rate, bpm	84.1 ± 19.9	84.3 ± 22.7	84.0 ± 16.9	0.942
	SBP, mmHg	129.9 ± 21.9	133.6 ± 22.1	126.2 ± 21.2	0.025
	DBP, mmHg	76.9 ± 14.5	77.5 ± 14.7	76.4 ± 14.4	0.622
	Atrial fibrillation, n (%)	67 (39.2)	43 (50.6)	24 (27.9)	0.003
	Hypertension, n (%)	133 (77.8)	74 (87.1)	59 (68.6)	0.005
	Diabetes, n (%)	69 (40.4)	36 (42.4)	33 (38.4)	0.642
	Smoking, n (%)	44 (25.7)	18 (21.2)	26 (30.2)	0.221
	Stroke, n (%)	63 (36.8)	34 (40.0)	29 (33.7)	0.430
Etiology					
	Ischemic heart disease, n (%)	127 (74.3)	64 (75.3)	63 (73.3)	0.861
	Prior MI, n (%)	91 (53.2)	38 (44.7)	53 (61.6)	0.032
	Cardiomyopathy, n (%)	19 (11.7)	3 (3.5)	16 (18.6)	0.003
	Other, n (%)	25 (14.6)	18 (21.2)	7 (8.1)	0.018
Laboratory results					
	WBC, ×${\mathrm{10}}^{9}$/L	7.3 ± 3.6	7.5 ± 4.5	7.1 ± 2.4	0.522
	Hb, g/L	129.2 ± 21.8	126.7 ± 21.2	131.7 ± 22.1	0.133
	Plt, ×${\mathrm{10}}^{9}$/L	198.4 ± 79.0	203.8 ± 87.3	193.1 ± 69.9	0.378
	HbA1C, %	6.9 ± 1.6	6.8 ± 1.4	7.0 ± 1.8	0.49
	Total protein, g/L	62.6 ± 7.6	62.9 ± 6.5	62.3 ± 8.5	0.595
	Albumin, g/L	37.6 ± 4.7	37.5 ± 4.9	37.8 ± 4.4	0.710
	FPG, mmol/L	7.2 ± 3.1	7.1 ± 2.9	7.3 ± 3.4	0.752
	ALT, U/L	26.9 ± 2.6	28.1 ± 3.2	25.8 ± 2.3	0.560
	Urea nitrogen, mmol/L	7.7 ± 3.9	7.7 ± 3.6	7.7 ± 4.1	0.875
	eGFR, mL/(min × 1.73 $m^{2}$)	73.5 ± 21.8	70.5 ± 20.0	76.6 ± 23.2	0.065
	Total-cholesterol, mmol/L	3.7 ± 1.2	3.8 ± 1.2	3.6 ± 1.1	0.508
	Triglycerides, mmol/L	1.3 ± 0.9	1.3 ± 0.8	1.2 ± 1.0	0.513
	LDL-C, mmol/L	2.1 ± 0.8	2.1 ± 0.9	2.1 ± 0.8	0.798
	HDL-C, mmol/L	1.1 ± 0.3	1.2 ± 0.3	1.1 ± 0.3	0.077
	Uric acid, umol/L	419.7 ± 156.4	409.8 ± 139.1	429.6 ± 172.0	0.410
	NT-proBNP, pg/mL ^a^	1980.0 (322.0, 35,000.0)	1290.0 (366.0, 35,000.0)	3135.0 (322.0, 35,000.0)	<0.001
Echocardiographic results					
	EF, %	49.7 ± 15.5	62.7 ± 8.0	36.9 ± 9.0	<0.001
	LAID, cm	4.6 ± 0.9	4.6 ± 0.9	4.7 ± 0.9	0.668
	LVID, cm	5.3 ± 0.9	4.7 ± 0.6	5.8 ± 0.9	<0.001
	RAID, cm	4.6 ± 1.1	4.7 ± 1,1	4.5 ± 1.0	0.454
	RVID, cm	2.5 ± 0.4	2.4 ± 0.4	2.5 ± 0.3	0.065
NYHA classification					
	II	132 (77.2)	74 (87.1)	58 (67.4)	0.003
	III	33 (19.3)	11 (12.9)	22 (25.6)	0.052
	IV	6 (3.5)	0 (0.0)	6 (7.0)	0.029
	DAPA, n (%)	51 (29.8)	24 (28.2)	27 (31.4)	0.739

Values are mean ± SD; ^a^, data were recorded as the median with IQR. Abbreviations: ALT, alanine aminotransferase; BMI, body mass index; bpm, beats per minute; DAPA, dapagliflozin; DBP, diastolic blood pressure; Drp1, dynamin-related protein 1; eGFR, estimated glomerular filtration rate; EF, left ventricular ejection fraction; FPG, fasting plasma glucose; Hb, hemoglobin; HDL-C, high-density lipoprotein-cholesterol; HFrEF, heart failure with reduced ejection fraction; HFpEF, heart failure with preserved ejection fraction; LAID, internal diameters of left atrium; LDL-C, low-density lipoprotein-cholesterol; LVID, internal diameters of left ventricle; MI, myocardial infarction; n, number; NYHA, New York Heart Association; NT-proBNP, N-terminal pro–B-type natriuretic peptide; Plt, platelet; RAID, internal diameters of right atrium; RVID, internal diameters of right ventricle; SBP, systolic blood pressure; WBC, white blood cell count.

### 3.2 The Diagnostic Value of Serum Drp1 in HF

**Fig. 2.**
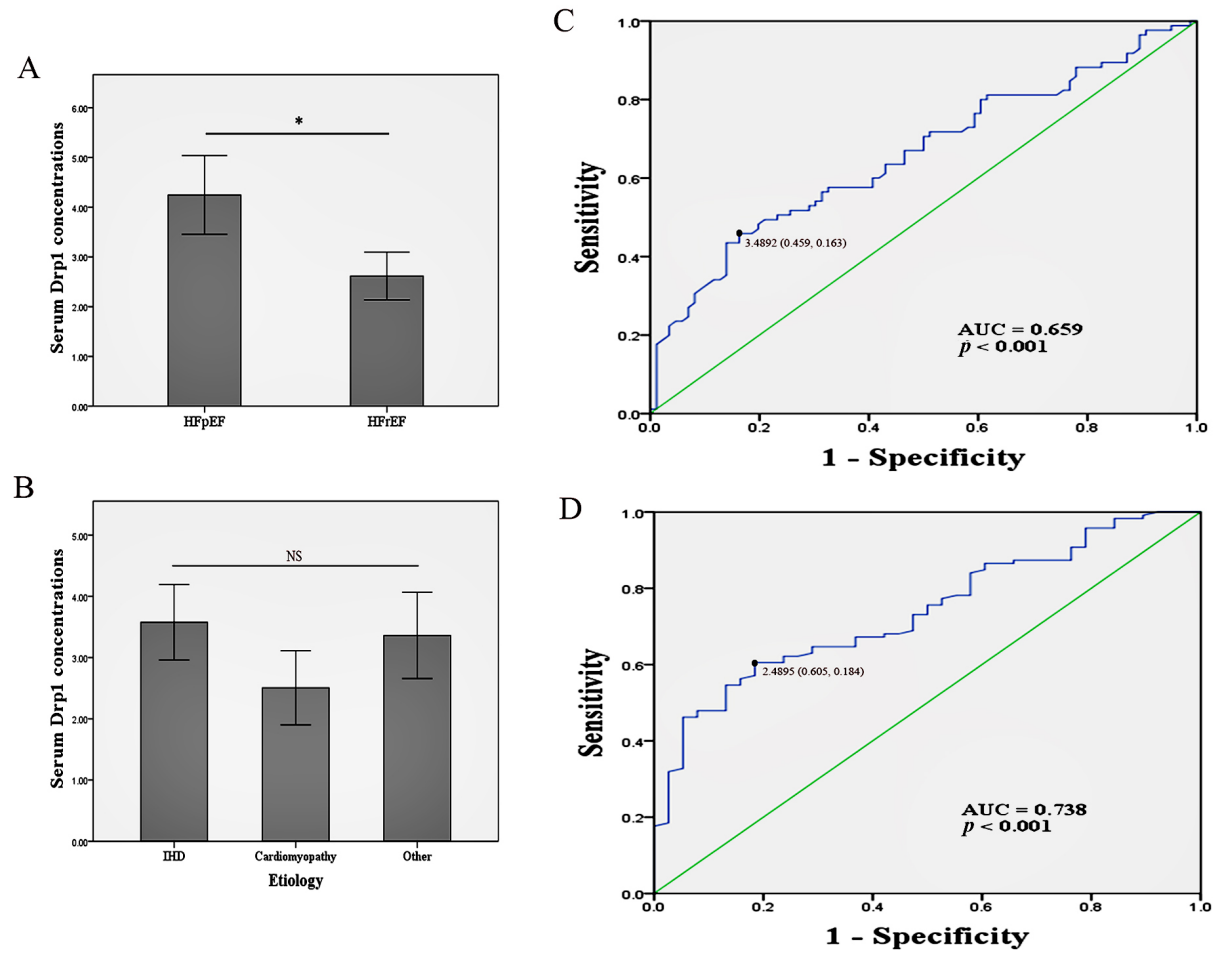
**Column graphs and the receiver operating characteristic (ROC) curves**. (A) Quantifications of serum Drp1 concentrations in patients of heart failure with preserved ejection fraction (HFpEF) and heart failure with reduced ejection fraction (HFrEF), respectively. *p < 0.05, Student’s *t*-test. (B) Quantifications of serum Drp1 concentrations in different etiologies of HF. IHD, ischemic heart disease; NS, no significance, one-way analysis of variance. (C) ROC curve for serum Drp1 in distinguishing HFpEF and HFrEF. (D) ROC curve for serum Drp1 to assess its indictive effects for freedom from the risk of composite endpoint at 6-month follow-up. The cutoff value of serum Drp1 is 2.5 ng/mL. AUC, area under the curve; Drp1, dynamin-related protein 1.

As shown in Fig. 2A, the serum Drp1 concentrations were significantly increased in the HFpEF group (4.2 ± 3.7 ng/mL vs. 2.6 ± 2.2 ng/mL, *p* = 0.001). We examined the serum Drp1 concentrations based on various etiologies of HF and found no significant difference between these groups (Fig. 2B), suggesting that the serum Drp1 concentrations were mainly dependent on the phenotypes of HF. An ROC curve for serum Drp1 was generated to distinguish HFpEF and HFrEF. As depicted in Fig. 2C, the optimal cutoff value was 3.5 ng/mL, with a sensitivity of 45.9% and specificity of 83.7% for the diagnosis of HFpEF. The area under the curve (AUC) was 0.659 [95% confidence interval (CI): 0.577–0.740, *p *< 0.001].

### 3.3 The Prognostic Value of Serum Drp1 for Patients with HF

**Fig. 3.**
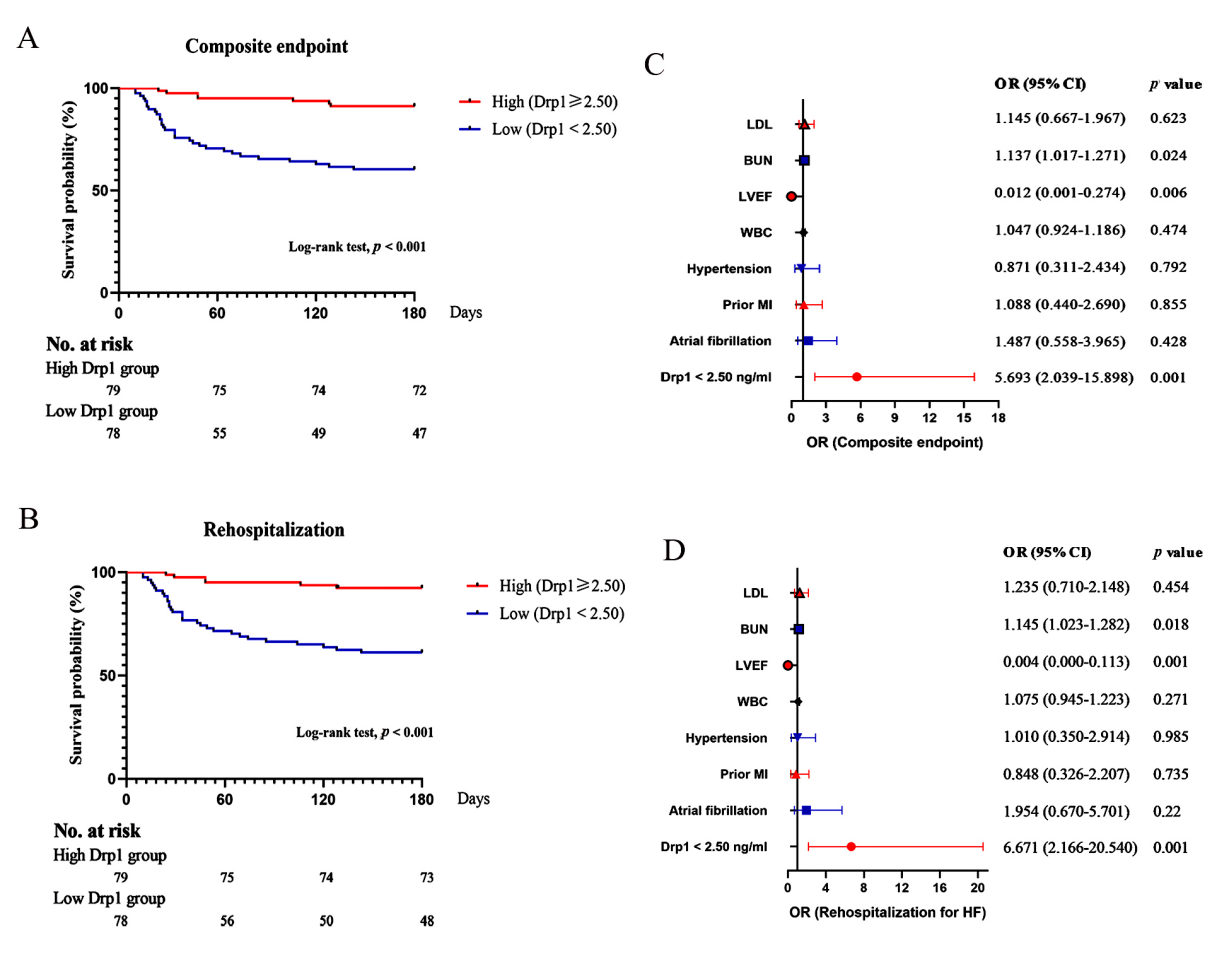
**Survival curves and forest plots**. (A,B) Kaplan-Meier curves for the composite endpoint (A) and rehospitalization for HF (B) in the low Drp1 group (Drp1 <2.5 ng/mL) versus the high Drp1 group (Drp1 ≥2.5 ng/mL). (C,D) Forest plots reveal the association between Drp1 at the threshold of >2.5 ng/mL and a composite endpoint (C) and rehospitalization for HF (D). Adjusted confounding factors included low-density lipoprotein cholesterol (LDL), WBC, hypertension, prior MI, and atrial fibrillation.

Seventy-seven patients with HFpEF and 80 patients with HFrEF completed the 6-month follow-up, and their clinical outcomes were collected for further analyses. Among these patients, none died during hospitalization, and 7 patients died after discharge (Table 2). According to the ROC curve analysis (Fig. 2D), the optimal cut-off value of serum Drp1 for freedom from the composite endpoint was 2.5 ng/mL, with a sensitivity of 60.5% and specificity of 81.6%. The AUC was 0.738 (95% CI: 0.656–0.820, *p *< 0.001). Accordingly, the patients were redivided into a high Drp1 group (serum Drp1 ≥2.5 pg/mL) and a low Drp1 group (serum Drp1 <2.5 pg/mL), and the baseline characteristics of these patients were listed in **Supplementary Table 1**. The results of the survival analysis indicated that a low serum concentration of Drp1 was associated with a higher risk of the composite endpoint (39.7% vs. 8.9%, *p *< 0.001, Fig. 3A), which was mainly driven by the increased incidence of rehospitalization for HF (38.5% vs. 7.6%, *p *< 0.001, Fig. 3B). Binary logistic regression analysis identified low concentrations of serum Drp1 [odds ratio (OR): 5.693, 95% CI: 2.039–15.898, *p* = 0.001], blood urea nitrogen (BUN) (OR: 1.137, 95% CI: 1.017–1.271, *p* = 0.024) and left ventricular ejection fraction (LVEF) (OR: 0.012, 95% CI: 0.001–0.274, *p* = 0.006) as the independent risk predictors for the composite endpoint after adjusting for confounding factors, including AF, prior MI, hypertension, white blood cell count (WBC), and low-density lipoprotein cholesterol (LDL) (Fig. 3C). These three predictors were also confirmed to be associated with an increased risk of rehospitalization for HF (low Drp1: OR: 6.671, 95% CI: 2.166–20.540, *p* = 0.001; BUN: OR: 1.145, 95% CI: 1.023–1.282, *p* = 0.018; LVEF: OR: 0.004, 95% CI: 0.000–0.113, *p* = 0.001, Fig. 3D).

**Table 2.** **Clinical follow-up in the Low Drp1 and High Drp1 groups**.

	1-month, n (%)			6-month, n (%)		
	Drp1 <2.5 (n = 78)	Drp1 ≥2.5 (n = 79)	*p*-value	Drp1 <2.5 (n = 78)	Drp1 ≥2.5 (n = 79)	*p*-value
Composite endpoint	15 (19.2)	2 (2.5)	0.001	31 (39.7)	7 (8.9)	<0.001
Rehospitalization for HF	14 (17.9)	2 (2.5)	0.001	30 (38.5)	6 (7.6)	<0.001
Cardia death	2 (2.6)	0 (0.0)	0.245	5 (6.4)	1 (1.3)	0.117
All-cause death	2 (2.6)	0 (0.0)	0.245	5 (6.4)	2 (2.5)	0.276

Abbreviations: Drp1, dynamin-related protein 1; HF, heart failure; MACEs, major adverse cardiac events; n, number.

## 4. Discussion

This observational study represents the first evaluation for the clinical values of serum Drp1. We found that serum Drp1 concentrations were much higher in HFpEF than in HFrEF (*p* = 0.001), and the ROC curve analysis indicated it could be a potential diagnostic biomarker for distinguishing the phenotype of HF (AUC = 0.659). When we combined the results of K-M survival analyses with the generated ROC curve of Drp1 for freedom from the composite endpoint, low serum concentrations of Drp1 (cut-off value = 2.5 ng/mL, AUC = 0.738) were found to be associated with a poor prognosis from HF. A low serum concentration of Drp1 was identified as an independent risk predictor for rehospitalization for HF (OR: 6.671, 95% CI: 2.166–20.540, *p* = 0.001), and led to a significantly increased risk of the composite endpoint. These findings suggested that low serum concentrations of Drp1 might serve as a biomarker for distinguishing HF phenotypes and the overall prognosis of HF, as well as providing a new potential therapeutic target for HF patients.

In adult cardiomyocytes, mitochondria account for about 30% of the total cell volume and produce vast amounts of ATP through oxidative phosphorylation to maintain contractile function [12]. HF commonly occurs with cardiac remodeling, in which there are significant molecular changes due to oxidative stress and myocyte loss through autophagy, including mitophagy, apoptosis, and fibrosis [17]. Thus, both the decrease in the number of contractile units and the damaged mitochondrial bioenergetic capacity in residual cardiomyocytes after myocardial injuries are directly linked with the progression of HF [18, 19]. The coordinated cycle of mitochondrial fission and fusion is known as mitochondrial dynamics, whose homeostasis has been demonstrated to have a critical role in maintaining cardiac structure and function [20, 21]. Drp1 is known as a crucial regulator of mitochondrial fission and is involved in mitophagy for degradation of depolarized mitochondria in the heart [22]. Parkin-dependent mitophagy is considered to be more critical for the maintenance of mitochondrial respiratory function in the absence of Drp1-dependent mitophagy [22]. In contrast, several other studies indicated that Parkin-dependent mitophagy would be hyper-activated in Drp1-deficient mouse hearts, which was thought to be detrimental to the heart because the downregulation of Drp1 induced constitutive recruitment of Parkin to the elongated mitochondria and increased degradation of healthy mitochondria [23, 24]. Based on the findings from these studies, Drp1 has been recognized as having an important role in affecting programmed cell death and cardiac metabolism through the mediation of mitophagy. Values of serum Drp1 may be an alternative way to determine myocardial damage compared to the more costly and invasive myocardial biopsy.

Our ROC curve analysis suggested that serum Drp1 can be a potential diagnostic biomarker for distinguishing HFpEF from HFrEF (AUC = 0.659), with a sensitivity of 45.9% and specificity of 83.7%. Currently, the diagnosis of HFpEF mainly depends on echocardiography findings. In clinical practice, the most commonly used biomarkers for the diagnosis of HF are plasma BNP or NT-proBNP levels, showing a much higher sensitivity (BNP at a threshold of ≤100 ng/L is 0.95, while NT-proBNP at a threshold of ≤300 ng/L is 0.99) but a relatively low specificity (BNP ≤100 ng/L is 0.63, and NT-proBNP ≤300 ng/L is 0.43), which may limit accurate risk stratification for HF [25, 26]. Although the sensitivity of serum Drp1 for the diagnosis of HFpEF is slightly lower compared to these classical markers, the specificity is much higher. A new concept of HF with improved EF has been raised by the latest ESC guidelines to provide more precise risk stratification of HF to optimize the clinical management of these patients [7]. Moreover, the NT-proBNP levels were much higher in these HF patients but showed no significant difference between the low and high Drp1 groups (2340.0 pg/mL vs. 1810.0 pg/mL, *p* = 0.126). Therefore, serum Drp1 combined with plasma BNP or NT-proBNP may provide more accurate definitions of HF phenotypes.

ROC curves were also generated for Drp1 to assess its role in determining freedom from the risk of the composite endpoint. The AUC was 0.738 (95% CI: 0.656–0.820, *p *< 0.001), and the optimal cut-off point was identified as 2.5 ng/mL. Combined with the results of the K-M survival analyses and binary logistic regression, low concentrations of Drp1 (Drp1 <2.5 ng/mL) were associated with poorer outcomes from HF and were identified as an independent risk predictor of rehospitalization for HF (OR: 6.671, 95% CI: 2.166–20.540, *p* = 0.001). The source of serum Drp1 remains unclear. Our prior study established an MI model in SD rats for 6 weeks and indicated that the decreased expression of Drp1 in the infarcted myocardium leads to structural and functional damage to cardiac mitochondria, which results in worse cardiac function [27]. The biological function of Drp1 mainly depends on its translocation from the cytoplasm to OMM to bind with the localized target genes [10, 11, 12]. Under these conditions, few Drp1 proteins would be released though large amounts of cardiomyocytes are ruptured. A prior study also demonstrated that Drp1-dependent mitochondrial autophagy would be transiently activated when stimulated by pressure overload, and the pathway was downregulated [28]. This may be a potential explanation for the clinical measurements of serum Drp1 found in this study. To date, no cell types have been identified for the secretion of Drp1 and further explorations for the source of serum Drp1 are still necessary.

In our study, we found no significant difference in mortality between the low and high Drp1 groups. HF patients usually die from a sudden cause (commonly recognized as a malignant arrhythmia) or from multiple organ dysfunction caused by end-stage respiratory and circulatory failure [29, 30]. In our study, IHD was confirmed as the main cause of HF, and more than half of the patients suffered from a prior MI. However, the serum Drp1 concentrations showed no significance between these groups divided by different etiologies of HF, suggesting Drp1 can be used to predict the prognosis of HF.

## 5. Limitation

Several limitations should be acknowledged in the current study. First, this is a single-center, observational study with a small sample size. Larger trials are warranted. Second, the potential regulation of oral agents on Drp1 could not be completely eliminated, especially with the use of Dapagliflozin (DAPA), which could regulate the expression level of Drp1 in the infarcted myocardium [27]. However, the baseline usage of DAPA showed no significant difference in this study. Third, longer follow-up is necessary for strengthening the association between serum Drp1 and the prognosis of HF. In addition, dynamic detection of Drp1 might help us better understand the variation of Drp1 along with changes in patient status. Finally, missing data of several inflammatory markers, including hypersensitive C-reactive protein and procalcitonin, also limited our ability to further explore their relevant effects on patient outcomes.

## 6. Conclusions

Our results indicated that serum Drp1 concentrations are significantly higher in patients with HFpEF versus those with HFrEF. It might serve as a good diagnostic marker for the distinction of HF phenotypes. A low serum concentration of Drp1 was identified as an independent risk predictor for poor clinical outcomes among these HF patients. In summary, serum Drp1 may serve as a meaningful biomarker to discriminate the diagnosis of HF phenotypes and the overall prognosis of HF, as well as become a potential therapeutic target for treating this disease.

## Data Availability

All data generated or analyzed during this study are included in this published article.

## Supplementary Material

Supplementary material associated with this article can be found, in the online version, at https://doi.org/10.31083/j.rcm2404123.
