# Supplementary material for: Serum Dynamin-Related Protein 1 Concentrations Discriminate Phenotypes and Predict Prognosis of Heart Failure
Source: Rev Cardiovasc Med. 2023 Apr 23;24(4):123. doi: 10.31083/j.rcm2404123 (PMC11273009; doi:10.31083/j.rcm2404123)
Supplement: Supplementary file 1 [file 2153-8174-24-4-123-s1.zip › 2153-8174-24-4-123-s1.docx]

**Supplementary table 1: Baseline characteristics between the low Drp1 and high Drp1 groups**

| **Variables** | **Total (n=171)** | **Drp1< 2.5 (n=84)** | **Drp1≥2.5 (n=87)** | ***p*-value** |
| --- | --- | --- | --- | --- |
| ***Demographics*** |  |  |  |  |
| Male, n (%) | 103 (60.2) | 54 (64.3) | 49 (56.3) | 0.349 |
| Age, years | 70.1±11.4 | 68.8±11.6 | 71.3±11.1 | 0.155 |
| BMI, kg/m^2^ | 25.8±4.8 | 25.4±4.7 | 26.2±5.0 | 0.249 |
| Heart rate, bpm | 84.1±19.9 | 82.9±20.0 | 85.3±20.0 | 0.430 |
| SBP, mmHg | 129.9±21.9 | 126.5±21.4 | 133.2±22.1 | 0.046 |
| DBP, mmHg | 76.9±14.5 | 75.8±14.5 | 78.0±14.5 | 0.328 |
| Atrial fibrillation, n (%) | 67 (39.2) | 27 (32.1) | 40 (46.0) | 0.085 |
| Hypertension, n (%) | 133 (77.8) | 61 (72.6) | 72 (82.8) | 0.141 |
| Diabetes, n (%) | 69 (40.4) | 36 (42.9) | 33 (37.9) | 0.536 |
| Smoking, n (%) | 44 (25.7) | 24 (28.6) | 20 (23.0) | 0.485 |
| Stroke, n (%) | 63 (36.8) | 33 (39.3) | 30 (34.5) | 0.530 |
| ***Etiology*** |  |  |  |  |
| Ischemic heart disease, n (%) | 127 (74.3) | 66 (78.6) | 61 (70.1) | 0.225 |
| Prior MI, n (%) | 91 (53.2) | 53 (63.1) | 38 (43.7) | 0.014 |
| Cardiomyopathy, n (%) | 19 (11.7) | 10 (11.9) | 9 (10.3) | 0.811 |
| Other, n (%) | 25 (14.6) | 8 (9.5) | 17 (19.5) | 0.083 |
| ***Laboratory results*** |  |  |  |  |
| WBC, x10^9^/L | 7.3±3.6 | 6.8±1.7 | 7.8±4.7 | 0.070 |
| Hb, g/L | 129.2±21.8 | 130.2±21.4 | 128.3±22.1 | 0.559 |
| Plt, x10^9^/L | 198.4±79.0 | 183.9±65.8 | 212.4±88.1 | 0.018 |
| HbA1C, % | 6.9±1.6 | 7.0±1.6 | 6.9±1.7 | 0.805 |
| Total protein, g/L | 62.6±7.6 | 62.9±5.7 | 62.2±9.1 | 0.516 |
| Albumin, g/L | 37.6±4.7 | 38.1±4.3 | 37.2±5.0 | 0.194 |
| FPG, mmol/L | 7.2±3.1 | 7.2±3.3 | 7.2±3.0 | 0.978 |
| ALT, U/L | 26.9±2.6 | 25.8±2.5 | 28.0±3.0 | 0.579 |
| Urea nitrogen, mmol/L | 7.7±3.9 | 8.3±4.5 | 7.1±3.0 | 0.041 |
| eGFR, ml/(min*1.73m^2^) | 73.5±21.8 | 73.4±23.0 | 73.7±20.8 | 0.926 |
| Total-cholesterol, mmol/L | 3.7±1.2 | 3.5±0.9 | 3.9±1.4 | 0.046 |
| Triglycerides, mmol/L | 1.3±0.9 | 1.3±1.0 | 1.3±0.9 | 0.624 |
| LDL-C, mmol/L | 2.1±0.8 | 1.9±0.6 | 2.3±1.0 | 0.011 |
| HDL-C, mmol/L | 1.1±0.3 | 1.1±0.3 | 1.1±0.3 | 0.901 |
| Uric acid, umol/L | 419.7±156.4 | 431.3±170.9 | 408.6±141.0 | 0.343 |
| NT-proBNP, pg/ml ^a^ | 1980.0 (322.0, 35000.0) | 2340.0 (322.0, 35000.0) | 1810.0 (339.0, 20400.0) | 0.126 |
| ***Echocardiographic results*** |  |  |  |  |
| EF, % | 49.7±15.5 | 45.7±14.6 | 53.6±15.4 | 0.001 |
| LAID, cm | 4.6±0.9 | 4.7±1.0 | 4.6±0.9 | 0.670 |
| LVID, cm | 5.3±0.9 | 5.4±1.0 | 5.1±0.8 | 0.011 |
| RAID, cm | 4.6±1.1 | 4.5±1,0 | 4.7±1.1 | 0.354 |
| RVID, cm | 2.5±0.4 | 2.5±0.3 | 2.5±0.4 | 0.797 |
| **NYHA classification** |  |  |  |  |
| II | 132 (77.2) | 61 (72.6) | 71 (81.6) | 0.202 |
| III | 33 (19.3) | 19 (22.6) | 14 (16.1) | 0.334 |
| IV | 6 (3.5) | 4 (4.8) | 2 (2.3) | 0.438 |
| DAPA, n (%) | 51 (29.8) | 27 (32.1) | 24 (27.6) | 0.616 |

Values are mean ± SD; a: data were recorded as the median with IQR;

**Abbreviations:** ALT, alanine aminotransferase; BMI, body mass index; bpm, beats per minute; DAPA, Dapagliflozin; DBP, diastolic blood pressure; Drp1, Dynamin-related protein 1; eGFR, estimated glomerular filtration rate; EF, left ventricular ejection fraction; FPG, fasting plasma glucose; Hb, hemoglobin; HDL-C, high-density lipoprotein-cholesterol; HFrEF, heart failure with reduced ejection fraction; HFpEF, heart failure with preserved ejection fraction; LAID, internal diameters of left atrium; LDL-C, low-density lipoprotein-cholesterol; LVID, internal diameters of left ventricle; MI, myocardial infarction; n, number; NYHA, New York Heart Association; NT-proBNP, N-terminal pro–B-type natriuretic peptide; Plt, platelet; RAID, internal diameters of right atrium; RVID, internal diameters of right ventricle; SBP, systolic blood pressure; WBC, white blood cell count;
